# Clinical profile of ocular trauma in a tertiary care hospital at Kolar district, South India

**DOI:** 10.6026/973206300213355

**Published:** 2025-09-30

**Authors:** Yeddula Venkata Rohith Reddy, Sangeetha T., Harshitha C.

**Affiliations:** 1 Department of Ophthalmology, Sri Devaraj Urs Medical College, Kolar, Karnataka, India

**Keywords:** Ocular trauma, eye injuries, epidemiology, clinical profile, visual outcome, road traffic accidents, closed-globe injury, open-globe injury, intraocular foreign body, optic nerve injury, ocular trauma score, prognostic factors, public health, preventive ophthalmology, tertiary care hospital, South India

## Abstract

Ocular trauma is a leading cause of preventable blindness worldwide. Hence, we retrospectively analyzed 350 cases at a tertiary hospital in South India (2021-2023) to profile injury patterns, outcomes and prognostic indicators. Most patients were young males, with road traffic accidents as the major cause. Closed-globe injuries predominated, while open-globe injuries carried poor prognosis. Significant visual improvement occurred in nearly half of cases; predictors of poor outcome included relative afferent pupillary defect, intraocular foreign body and optic nerve injury. Thus, strengthened preventive strategies and timely interventions are critical to reducing trauma-related blindness.

## Background:

Ocular trauma remains a major public health challenge, accounting for up to 5% of global blindness [1]. Worldwide, over 55 million eye injuries occur annually, with 1.6 million leading to permanent blindness [2]. Incidence rates range 8-14 per 100,000 populations [3]. In India, ocular trauma prevalence is reported at 0.6%-2%, disproportionately affecting young adult males due to occupational exposure and road traffic risks [4]. Road traffic accidents (RTAs) are consistently identified as the leading etiology [5]. Prognosis depends on injury severity and timeliness of management [6]. COVID-19 modified trauma epidemiology, with increased domestic injuries despite reduced hospital admissions [7, 8]. Closed-globe injuries are most frequent, though open-globe injuries have higher risk of endophthalmitis and retinal detachment [9, 10]. Prognostic determinants include relative afferent pupillary defect (RAPD), vitreoretinal involvement and optic nerve trauma [11]. Scoring systems such as the Ocular Trauma Score (OTS) and Pediatric Ocular Trauma Score (POTS) aid prognostication and patient counseling [12, 13]. Imaging modalities include B-scan ultrasonography and CT orbit enhance triage and surgical planning [14]. Despite advances, regional Indian data remain sparse, especially from South India [15]. This study profiles ocular trauma cases in a tertiary center, focusing on etiology, injury type, clinical findings, outcomes and prognostic markers. Therefore, it is of interest to describe and bridge the gap in regional ocular trauma data and provide updated evidence from a tertiary hospital in South India.

## Materials and Methods:

## Design:

Retrospective observational study.

## Setting:

Department of Ophthalmology, R.L. Jalappa Hospital, Kolar, Karnataka.

## Period:

January 2021 - December 2023.

## Sample:

350 patients (exceeding calculated minimum of 98).

## Inclusion:

All documented trauma cases during study period.

## Exclusion:

Incomplete records, pre-existing non-trauma ocular pathology.

## Data:

Demographics, etiology, injury type, clinical and radiological findings, management, outcomes.

## Analysis:

SPSS v22. Descriptive statistics. Chi-square/t-tests for associations. p < 0.05 significant.

## Ethics:

Institutional Ethics Committee approval (SDUAHER/KLR/R & D/CEC/S/PG/71/2024-25).

## Results:

A total of 350 patients with ocular trauma were analyzed. The mean age was 31.8 ± 14.2 years, with a clear male predominance (77.1%), and the most commonly affected age group was 21-40 years (54.8%) (Figure 1 - see PDF). Road traffic accidents (RTAs) were the leading cause, accounting for 60.6% of injuries, followed by occupational hazards (19.7%), domestic accidents (11.4%), assaults (5.1%), and sports-related injuries (3.1%) (Figure 2 - see PDF). Closed-globe injuries were the most frequent type (82.6%), with open-globe injuries (10.3%) and chemical/burn injuries (7.1%) being less common (Figure 3 - see PDF). Common clinical findings included lid laceration (22.6%), corneal abrasion (18.3%), hyphema (12.8%), relative afferent pupillary defect (11.1%), lens dislocation (8.0%), retinal detachment (5.4%), and intraocular foreign body (4.9%). Radiological evaluation revealed orbital fractures in 15.7% (predominantly inferior wall) and optic nerve injury in 3.1% of cases. At presentation, only 11.1% had visual acuity ≥ 6/18, while 65.7% presented with vision < 6/60. After treatment, 46.2%achieved ≥ 6/18, with a significant overall improvement (p < 0.001) (Figure 4 - see PDF). Predictors of poor visual outcome included RAPD, optic nerve trauma, and intraocular foreign body (p < 0.05) (Figure 5 - see PDF).

## Discussion:

This study highlights ocular trauma as a predominant ophthalmic emergency affecting males in their productive years, consistent with global and Indian literature [1, 4]. RTAs accounted for >60% of cases, mirroring national data emphasizing rapid urbanization, poor traffic enforcement and low helmet compliance [5, 7]. A recent multi-center South Indian analysis reported RTAs as the single most common cause of ocular injuries, underscoring the critical need for stricter helmet compliance, road safety enforcement, and public awareness initiatives across India [16]. Similarly, Wagh *et al.* observed that 56.7% of ocular trauma cases were attributable to RTAs in a rural central Indian population, with a striking male preponderance of nearly 88% [17]. This highlights that our findings mirror other rural Indian cohorts. Closed-globe injuries dominated (82.6%), aligning with previous South Indian studies [15]. Although less severe than open-globe injuries, closed-globe trauma can still impair vision when involving the posterior segment. Open-globe injuries carried higher risks of endophthalmitis, retinal detachment and globe loss, as previously documented [6, 10]. Visual prognosis improved markedly with intervention: nearly half achieved ≥6/18 vision post-treatment. However, poor outcomes were strongly associated with RAPD, IOFB and optic nerve trauma. These findings reinforce the prognostic value of OTS, validated by Agrawal *et al.* and Kuhn *et al.* [6, 13]. Radiological tools (B-scan, CT orbit) were invaluable in detecting posterior and orbital injuries, consistent with Zha *et al.* [14]. The COVID-19 era reshaped trauma epidemiology. Halawa *et al.* reported increased domestic accidents despite reduced hospital attendances, paralleling shifts observed in our dataset [8].

## Strengths:

Large sample size, comprehensive analysis of mechanisms and prognostic factors.

## Limitations:

Retrospective design, absence of long-term follow-up, lack of functional/quality-of-life assessment.

## Public health implications:

Urgent emphasis on helmet enforcement, workplace safety, trauma registries and rapid referral networks. Adoption of prognostic scoring systems should be routine to guide management and counseling.

## Conclusion:

Ocular trauma, dominated by RTA-related closed-globe injuries, is a leading cause of visual morbidity in South India. Timely intervention and prognostic assessment significantly improve outcomes. Hence, preventive strategies including strict helmet laws, workplace safety measures and structured trauma registries are essential to reduce trauma related blindness.
